# Preparation of Glass-Ceramic Materials by Controlled Crystallization of Eu_2_O_3_-Doped WO_3_-B_2_O_3_-La_2_O_3_ Glasses and Their Luminescent Properties

**DOI:** 10.3390/molecules30040832

**Published:** 2025-02-11

**Authors:** Aneliya Yordanova, Margarita Milanova, Lyubomir Aleksandrov, Reni Iordanova, Peter Tzvetkov, Pavel Markov, Petia Petrova

**Affiliations:** 1 Institute of General and Inorganic Chemistry, Bulgarian Academy of Sciences, G. Bonchev, Str., bld. 11, 1113 Sofia, Bulgaria; a.yordanova@svr.igic.bas.bg (A.Y.); lubomirivov@gmail.com (L.A.); reni@svr.igic.bas.bg (R.I.); tzvetkov@svr.igic.bas.bg (P.T.); pvlmarkov@svr.igic.bas.bg (P.M.); 2 Institute of Optical Materials and Technologies “Acad. Jordan Malinowski”, Bulgarian Academy of Sciences, blvd. Akad. G. Bonchev 109, 1113 Sofia, Bulgaria; petia@iomt.bas.bg

**Keywords:** glass-ceramics, XRD, TEM, luminescence

## Abstract

In this paper, the crystallization behavior of 52WO_3_:22B_2_O_3_:26La_2_O_3_:0.5Eu_2_O_3_ glass has been investigated in detail by XRD and TEM analysis. The luminescent properties of the resulting glass-ceramics were also investigated. By XRD and TEM analysis, crystallization of β-La_2_W_2_O_9_ and La_2_WO_6_ crystalline phases has been proved. Photoluminescent spectra showed increased emission in the resulting glass-ceramic samples compared to the parent glass sample due to higher asymmetry of Eu^3+^ ions in the obtained crystalline phases, where the active Eu^3+^ ions are incorporated. Also, in the glass-ceramics, the crystalline particles are embedded in the amorphous matrix and more of them are separated from each other which improves the light scattering intensity from the free interfaces of the nanocrystallites, resulting in the enhancement of the PL intensity. It was established that the optimum emission intensity is registered for glass-ceramic samples obtained after an 18 h heat treatment of the parent glass. After 21 h of glass crystallization, the amount of crystallite particles is high enough, and they are in close proximity to each other, and hence, the average distance between europium ions decreases, resulting in quenching of Eu^3+^ and a decrease in the emission intensity. Additionally, at 21 h of glass crystallization, formation of new crystalline phase—La_2_WO_6_ is established. A redistribution of Eu^3+^ ions in the different crystalline compounds is most likely taking place, which is also not favorable for the emission intensity.

## 1. Introduction

In recent years, rare earth-based tungstates have gathered increased attention as host materials due to their distinct crystal structure, remarkable emission properties, strong near-ultraviolet (NUV) absorption, thermal and chemical stability, low hygroscopicity, environmental friendliness, and self-activating luminescence properties. In addition, tungstates exhibit a broad charge transfer band (CTB) in the range of 200–350 nm. The CTB can transfer energy to rare earths, making them ideal for UV excitations [1]. In fact, rare earth tungstates are a big family including various compounds of RE_2_W_3_O_12_, RE_2_WO_6_, RE_6_WO_12_, RE_2_W_2_O_9_, RE_10_W_22_O_81_, and so forth, according to W/RE stoichiometry, and can be viewed as pseudo-binary compositions between RE_2_O_3_ and WO_3_ [2,3]. Among the rare earth tungstates, the compounds of La and tungsten are of great potential for luminescent host materials. For example, La_2_W_2_O_9_:Eu^3+^ shows promising photoluminescence and has a quantum yield as high as 77% [3,4,5,6]. La_2_(WO_4_)_3_:Tb^3+^ phosphors were successfully synthesized via an optimized microwave-assisted co-precipitation technique [7]. The luminescence characteristics and structural analyses of Sm^3+^-activated La_10_W_22_O_81_ microphosphors were also reported [1]. Eu^3+^:La_10_W_22_O_81_ (LWO) microphosphor rods with intense red emission were synthesized using a hydrothermal-assisted solid-state method [8]. To the best of our knowledge, there are no data for the preparation of lanthanum tungstate glass-ceramics by controlled glass crystallization. In recent years, a great deal of attention has been paid to rare earth-doped glass-ceramic materials, which play a crucial role in many optical applications such as up-conversion fibers, solid-state lasers, medical sensors, optical electronic chips, luminescence labels, optical amplifiers, 3D displays, etc. [9,10]. Glass-ceramics possess excellent characteristics found in both glasses and crystals and have none of the disadvantages of these two materials. Similar to glasses, glass-ceramics have a large capacity for accommodating an active rare earth dopant, are isotropic, and have evenly distributed activators within their bodies. Similar to single crystals, glass-ceramics contain rare earth ions within strictly ordered ligand surroundings. As a result, the presence of a crystalline environment around a rare earth ion allows for high absorption and emission cross-section reduction in the non-radiative relaxation process because of the lower phonon cut-off energy and tailoring of the ion–ion interaction by control of the rare earth ion partition [9,11]. The glass-ceramic materials are usually obtained by subsequent thermal treatment of a glass, which is first melted and annealed as usual. This conventional method relies on thermally induced phase separation and in situ crystallization processes, which are, however, very complex to experimentally control [11]. The choice of an appropriate glass composition is very important for luminescent glass-ceramics elaboration. The search for more efficient glass compositions and guiding structures for rare earth-doped glass-ceramics continues. In our earlier paper, we determined the glass formation region in the ternary La_2_O_3_-B_2_O_3_-WO_3_ system. It was established that transparent colorless glasses are obtained in the central part of the system between 20 and 35 mol% La_2_O_3_ [12]. We have also studied their structure and crystallization behavior. The main crystallization mechanism in the glasses is the surface crystallization, and the glass in mol % of 50WO_3_:25La_2_O_3_:25B_2_O_3_ is crystallized as a single phase LaBWO_6_ [13]. The luminescence properties of an Eu^3+^ doped glass, glass-crystalline product, and polycrystalline sample with the same composition of 50WO_3_:25La_2_O_3_:25B_2_O_3_ have been verified [12]. It was found that the intensity of emission increases drastically in the glass-crystalline sample consisting of LaBWO_6_:Eu^3+^ nanocrystals compared with the glass and polycrystalline samples. These results provoked our interest in continuing the investigation into an La_2_O_3_-B_2_O_3_-WO_3_ system. The purpose of the present study is to investigate the crystallization behavior of one selected glass composition from the isothermal section LaBWO_6_-La_2_W_2_O_9_ of the phase diagram [14] in order to check for the possibility of obtaining lanthanum tungstate glass-ceramics doped with Eu^3+^ ions and to study their photoluminescence properties. The crystallization behavior of glass-ceramics was characterized by X-ray diffraction and transmission electron microscopy. Emission spectra were measured, and color coordinates of the materials were determined.

## 2. Results and Discussion

### 2.1. XRD Data and DTA Analysis

**Figure 1:**
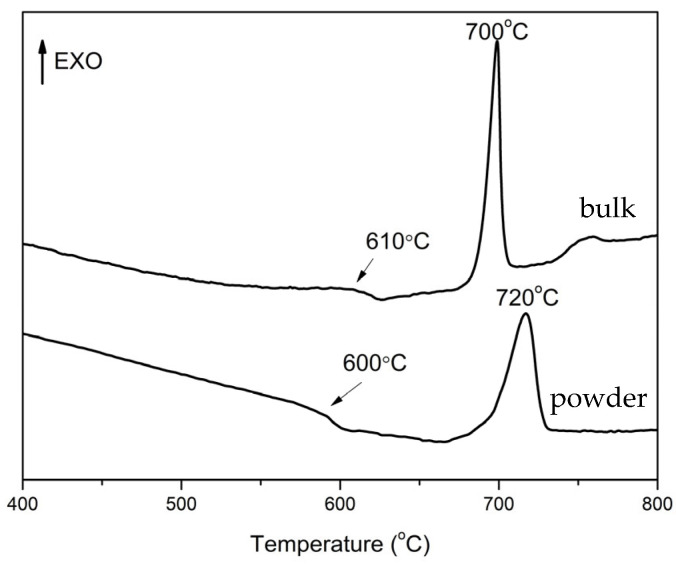
DTA curves of 52WO_3_:22B_2_O_3_:26La_2_O_3_:0.5Eu_2_O_3_ glass.

The DTA curves for the bulk and powder samples of 52WO_3_:22B_2_O_3_:26La_2_O_3_:0.5Eu_2_O_3_ glass at a heating rate of 10 K/min are shown in Figure 1. An endothermic dip for the glass transition and exothermic peak for crystallization are clearly observed in both samples. The values of the glass transition and crystallization peak temperatures are T_g_ = 610 °C and T_c_ = 700 °C for the bulk sample, and T_g_ = 600 °C and T_c_ = 720 °C for the powder glass sample. The difference in the T_c_ values of bulk and powder 52WO_3_:22B_2_O_3_:26La_2_O_3_:0.5Eu_2_O_3_ glass implies that a surface crystallization is taking place in this glass. The glass-ceramics (GCs) have been prepared by heat treatment of the precursor glass 52WO_3_:22B_2_O_3_:26La_2_O_3_:0.5Eu_2_O_3_ at 680 °C, which is the temperature at the beginning of the glass crystallization peak in the DTA curve of the bulk glass sample for 3 h, 6 h, 9 h, 15 h, 18 h, and 21 h.

**Figure 2:**
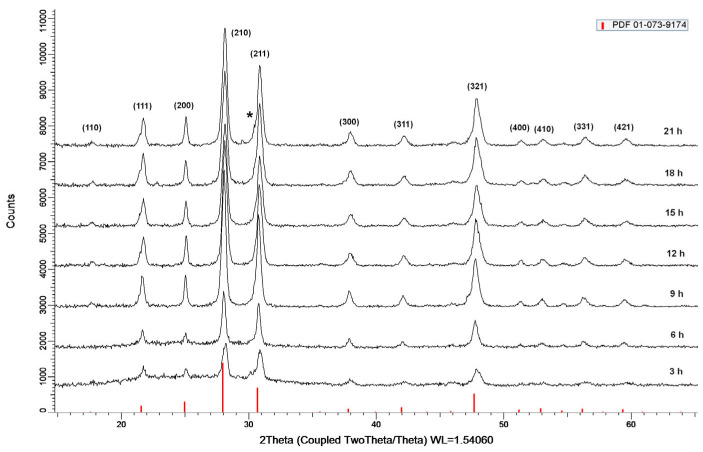
XRD patterns of 52WO_3_:22B_2_O_3_:26La_2_O_3_:0.5Eu_2_O_3_ glass ceramics crystallized at 680 °C with different durations. Indexed peaks represent the La_2_W_2_O_9_ cubic phase. For comparison, vertical red bars correspond to Bragg peaks of β-La_2_W_1.7_Mo_0.3_O_9_. Asterisk (*) represents the most intensive (312) peak of orthorhombic La_2_WO_6_ (PDF # 00-057-1075).

Figure 2 shows the powder diffraction patterns after crystallization of glass with composition 52WO_3_:22B_2_O_3_:26La_2_O_3_:0.5Eu_2_O_3_ at a temperature of 680 °C and a crystallization time of 3, 6, 9, 12, 15, 18, and 21 h, respectively. The glass-ceramics obtained after heat treatment of the parent glass for different lengths of time are designated as GC-3 h, GC-6 h, GC-9 h, GC-12 h, GC-15 h, GC-18 h, and GC-21 h, respectively.

From the phase analysis performed, the best match was found to be β-La_2_W_1.7_Mo_0.3_O_9_ (PDF # 01-073-9174), which is isostructural to the high-temperature polymorphic modification of La_2_Mo_2_O_9_ and crystallizes in cubic space group P2_1_3 with parameter a = 7.1327 Å [15]. After 21 h of glass crystallization, the appearance of an additional crystalline phase of orthorhombic La_2_WO_6_ was observed. To the best of our knowledge, no La_2_W_2_O_9_ with a cubic structure at room temperature has been reported so far.

Lanthanum molybdate is known to have two polymorphic modifications: an α-La_2_Mo_2_O_9_ low-temperature monoclinic form that is stable below 580 °C and a β-La_2_Mo_2_O_9_ cubic phase that is stable above this temperature [16]. Similarly, lanthanum tungstate also exhibits a reversible polymorphic transition at 1070 °C: a triclinic low-temperature α-La_2_W_2_O_9_ form and a cubic high-temperature β-La_2_W_2_O_9_ polymorph [17]. It is assumed that cubic lanthanum tungstate is isostructural to β-La_2_Mo_2_O_9_. According to our literature review, a crystal structure for β-La_2_W_2_O_9_ has not yet been published. Generally, the stabilization at room temperature of the high-temperature polymorphic modification of La_2_Mo_2_O_9_ and La_2_W_2_O_9_ is carried out with suitable cationic substitutions [18], and only β-La_2_W_1.7_Mo_0.3_O_9_ is reported as a single stable phase at room temperature without a reversible phase transition [15]. Based on this, we suggest that in our study, the isomorphous substitution of La^3+^ by Eu^3+^ has an influence on the stabilization of the cubic symmetry phase of La_2_W_2_O_9_. Moreover, in the chemical composition of the resulting glass, there are no other possibilities for isomorphic substitutions in the structure of β-La_2_W_2_O_9_.

Table 1 presents the calculated unit cell parameters and crystallite size for all stages of the crystallization of cubic β-La_2_W_2_O_9_. No significant change in unit cell parameters as a function of crystallization time is observed from the data. The small variations observed are most likely due to the conditions during powder pattern collection. This study was conducted on a bulk sample of initial glass, which inevitably has some surface irregularities and leads to systematic errors during X-ray diffraction. The calculated crystallite size also remains within narrow limits, between 27.8(6) nm and 22.1(4) nm, regardless of the crystallization time. The reasons for this are most likely kinetic, with the limiting factor in this case being the relatively low crystallization temperature of 680 °C.

### 2.2. Luminescent Properties

**Figure 3:**
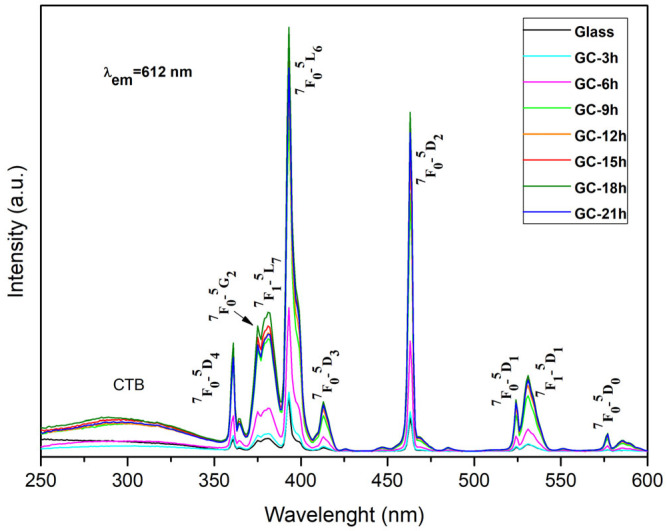
Excitation spectra of 0.5% Eu^3+^-doped 52WO_3_:22B_2_O_3_:26La_2_O_3_ glass and glass-ceramic samples.

To investigate the effect of heat treatment (level of crystallinity) on luminescent properties, we compared the excitation and emission spectra of 0.5% Eu^3+^-doped 52WO_3_:22B_2_O_3_:26La_2_O_3_ glass with those of the corresponding glass-ceramic (GC) samples obtained at different times of heat treatment at 680 °C. The excitation spectrum (Figure 3) monitored at a 612 nm wavelength (^5^D_0_ → ^7^F_2_ transition of Eu^3+^ ions) contains a continuous excitation band below 350 nm and several sharp peaks in the range from 350 nm to 600 nm at 361 nm, 374 nm 381 nm, 392 nm, 413 nm, 462 nm, 523 nm, 530 nm, and 576 nm, which corresponds to characteristic 4f—4f transitions of the Eu^3+^ ion from ^7^F_0_ → ^5^D_4_, ^7^F_0_ →^5^G_2_, ^7^F_0_ → ^5^L_7_, ^7^F_0_ → ^5^L_6_, ^7^F_0_ → ^5^D_3_, ^7^F_0_ → ^5^D_2_, ^7^F_0_ → ^5^D_1_, ^7^F_1_ → ^5^D_1_, and ^7^F_0_ → ^5^D_0_, respectively [19].

Among the above transitions, the ^7^F_0_ → ^5^L_6_ one, centered at 392 nm, stands out as the most prominent. Therefore, subsequent emission spectrum measurements were conducted under excitation at 392 nm. The wide band peaking at about 290 nm is assigned to the ligand-to-metal charge transfer states (LMCT) which appeared due to the combined effect of the electronic transition from the 2p orbital of O^2−^ ions to the vacant 4f energy level of the central Eu^3+^ ions in the Eu—O polyhedral and O^2-^ → W^6+^ in the WO_n_ groups (WO_n_ = WO_4_ and WO_6_) [20,21,22,23,24].

The presence of WO_n_ groups’ excitation band, recorded at the characteristic Eu^3+^ emission wavelength of 612 nm, suggests that non-radiative energy transfer from the glass matrix to the active rare-earth ion is occurring [24,25]. This mechanism is called host-sensitized luminescence.

**Figure 4:**
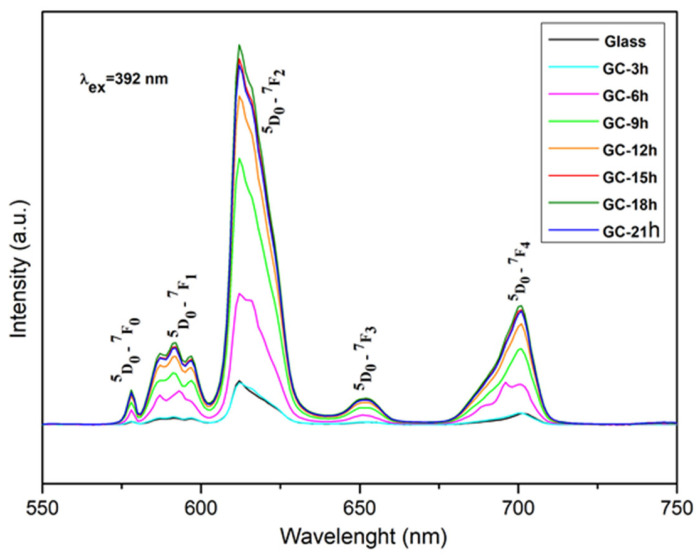
Emission spectra of 0.5% Eu^3+^-doped 52WO_3_:22B_2_O_3_:26La_2_O_3_ glass and glass-ceramic samples.

As can be seen from Figure 3, the excitation gradually increases with the increase in heat treatment time and reaches a maximum at 18 h and then decreases at 21 h. The same trend is observed in the emission spectra (Figure 4). This enhancement is most likely attributed to the progressive increase in the amount of the crystalline phase in the samples, which alters the crystal field environment inside the matrix, and hence, more efficient excitation of Eu^3+^ can be expected in the glass-ceramic samples.

Additionally, when comparing the intensity of LMCT and the f-f transitions, it is evident that the intensity is stronger for narrow Eu^3+^ bands. This is helpful for attaining proper excitation by near-UV and blue LED chips, as the intensity of these Eu^3+^ transitions is generally weak because they are forbidden by Laporte’s selection rule [26]

The emission spectra in Figure 4 show the conventional emission lines of Eu^3+^, which corresponds to the transitions from the ^5^D_0_ excited energy level towards the ^7^F_0_, ^7^F_1_, ^7^F_2_, ^7^F_3_, and ^7^F_4_ ground states at about 578, 592, 612, 651, and 700 nm, respectively.

The glass sample possesses the lowest emission intensity, and after obtaining glass-ceramic samples by heat treatment at 680 °C and crystallization of the La_2_W_2_O_9_ phase, the intensity increases up to 18 h of thermal treatment. This enhancement is most likely attributed to the increase in the crystallinity of the sample and due to the incorporation of Eu^3+^ in the obtained crystal phase.

It is well known that the distorting of the local environment around Eu^3+^ ions in the host lattice can improve the emission. In particular, the increased photoluminescence emission in glass-ceramics samples compared to the glass is related to the covalency and structural changes in the vicinity of Eu^3+^ ions (short range effect) [27]. The enhancement of the emission intensity is one proof that site symmetry around the active ion modifies with the glass → crystal transition. On the other hand, in the glass-ceramics, the crystalline particles are embedded in the amorphous matrix and more of them are separated from each other which improves the light scattering intensity from the free interfaces of the nanocrystallites [13], resulting in the increase in the PL intensity.

Additionally, when holding the heat treatment time from 6 h to 9 h, the emission intensity increased by approximately twofold, which is a result of the significant increase in the amount of crystalline phase on the surface (see Figure 2).

After 21 h of glass crystallization, the amount of crystallite particles is high enough and they are in close proximity to each other, and hence, the average distance between europium ions decreases, resulting in quenching of Eu^3+^ luminescence. As a result of the reduced amount of the amorphous phase on the surface, the effect of light scattering is also reduced, leading to a decrease in emissions. Additionally, at 21 h of glass crystallization, the formation of the new crystalline phase, La_2_WO_6_, is established. A redistribution of Eu^3+^ ions in the different crystalline compounds is most likely taking place, which is also not favorable for the emission intensity. The X-ray structural data and TEM analysis support these observations.

It is established that the ratio of ^5^D_0_ → ^7^F_2_ to ^5^D_0_ → ^7^F_1_ transitions may provide structural hints about the distortion of the crystal field environment around the rare earth ions. The ^5^D_0_ → ^7^F_1_ transition is a magnetic dipole (MD) transition among Eu^3+^ emission spectra and is permitted by Jude–Offelt theory. Its intensity is not influenced by the environment near Eu^3+^ ions. In contrast, the ^5^D_0_ → ^7^F_2_ transition is an electric dipole transition (ED), and its intensity is strongly dependent on the ligand environment. The dominance of the ^5^D_0_ → ^7^F_2_ transition over the ^5^D_0_ → ^7^F_1_ transition in the emission spectra (Figure 4) is an indication that the environment surrounding Eu^3+^ in the samples is non-centrosymmetric. In particular, the site symmetry of Eu^3+^ can be evaluated by using the integrated emission intensity ratio (R) of these two transitions ^5^D_0_ → ^7^F_2_/^5^D_0_ → ^7^F_1_. Higher values of R indicate more site asymmetry of the rare-earth ion, high covalency between Eu^3+^ and O^2-^ ions, increased emission intensity, and, hence, greater potential for optical applications [16,17,28].

The calculated intensity ratios R (from 4.88 to 6.12) of the Eu^3+^-doped 52WO_3_:22B_2_O_3_:26La_2_O_3_:0.5Eu_2_O_3_ glass and glass-ceramics (Table 2) are mostly higher than other reported Eu^3+^ oxide compositions [29,30,31,32,33,34,35,36]. It is also evident from Table 2 that the asymmetry ratio of the glass-ceramics is higher than that of glass and progressively rises with increasing heat treatment time of the samples up to 18 h (GC-18 h), which demonstrates that the degree of symmetry of Eu^3+^ ions decreases, and a strong Eu-O covalence is formed at a higher level of crystallinity. The larger the asymmetry, the greater the luminous intensity, and this fact is directly related to the higher emission intensity of glass-ceramics. This result is consistent with the intensity trend in PL spectra, where the intensity increases with an increase in the crystallinity level. At 21 h of heat treatment (CG-21), a decrease in the R value is observed, which agrees with the decrease in the emission intensity of this specimen.

Additionally, the appearance of the sensitivity to the crystal field and the forbidden ^5^D_0_ → ^7^F_0_ transition based on the standard Judd–Ofelt theory [37] indicates that the Eu^3+^ ion occupies non-centrosymmetric sites with C_2ν_, C_n_, or C_s_ symmetry [38].

**Figure 5:**
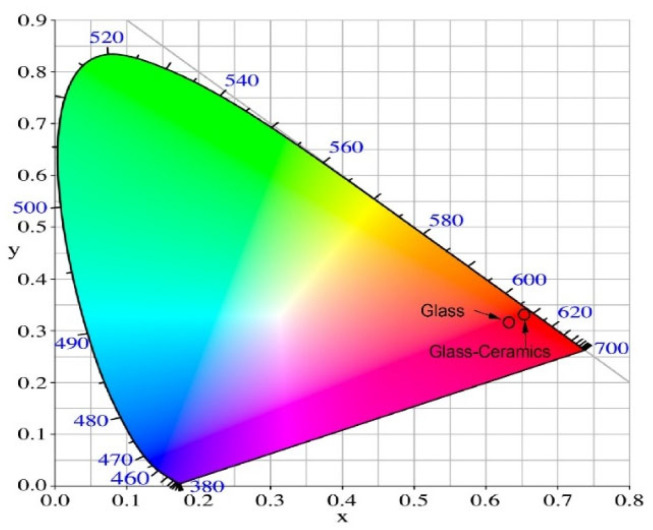
CIE chromaticity diagram of Eu^3+^-doped 52WO_3_:22B_2_O_3_:26La_2_O_3_:0.5Eu_2_O_3_ glass and corresponding glass-ceramics.

The Commission International de I’Eclairage (CIE) 1931 chromatic diagram is a standardized method for characterizing colors of emission. The chromaticity coordinates of Eu^3+^-doped 52WO_3_:22B_2_O_3_:26La_2_O_3_:0.5Eu_2_O_3_ glass and glass-ceramics in Table 3 are calculated from the emission spectra by using the color calculator software SpectraChroma Version 1.0.1. (CIE coordinate calculator) [39] and are illustrated in Figure 5. It can be observed that they are located in the red region of the diagram. The estimated coordinates of glass-ceramic samples with average values of 0.650 and 0.345 are represented in the diagram as one point, as the values are almost identical and are very similar to the CIE coordinate of the commercial red phosphor Y_2_O_2_S:Eu^3+^ (0.658; 0.340) [40]. The obtained results show that the investigated glass and glass-ceramics have the potential to be used as red-emitting materials.

### 2.3. TEM Investigations

**Figure 6:**
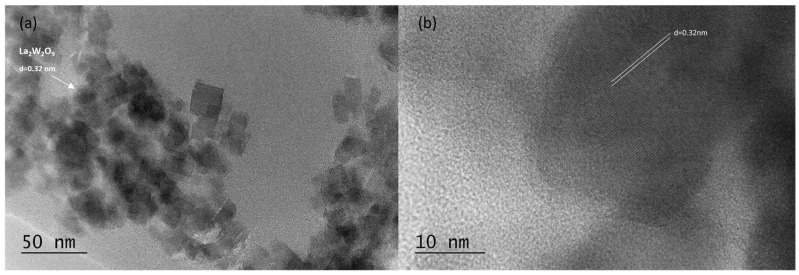
Bright field micrograph (**a**) and HRTEM (**b**) of nanosized particles of La_2_W_2_O_9_.

**Figure 7:**
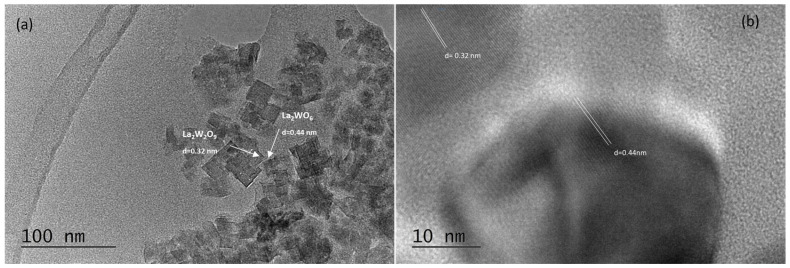
Bright field micrograph (**a**) and HRTEM (**b**) of La_2_W_2_O_9_ and La_2_WO_6_.

**Figure 8:**
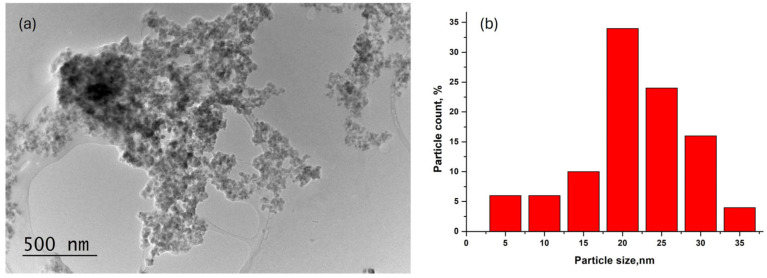
Bright field micrograph (**a**) and particle size distribution (**b**) of GC-18 h.

A glass-ceramic sample obtained after an 18 h thermal treatment of the parent glass, which exhibited the most intensive luminescent emission, was analyzed using TEM and HRTEM techniques. Morphological studies (Figure 6, Figure 7 and Figure 8) revealed that the sample contains spherical and rectangular nanosized particles. The inset in Figure 7 illustrates the particle size distribution. The nanosized particles range between 10 and 80 nanometers, with the majority falling within the 20–40 nm range, although some larger particles were also observed. The average particle size of the glass-ceramic sample was determined to be 22 nm.

The HRTEM results align well with those obtained from X-ray phase analysis. HRTEM analysis (Figure 6 and Figure 7) identified the presence of cubic LaW_2_O_9_ and orthorhombic La_2_WO_6_ phases in the sample. The cell parameters were measured as a = 7.33 Å for the cubic phase and a = 8.886 Å, b = 16.555 Å, and c = 5.521 Å for the orthorhombic phase. The corresponding interplanar distances were determined to be d = 3.2 Å and d = 4.4 Å, respectively.

## 3. Materials and Methods

### 3.1. Samples Preparation

Glass with the nominal composition 52WO_3_:22B_2_O_3_:26La_2_O_3_:0.5Eu_2_O_3_ was obtained by applying the conventional melt-quenching method, using commercial powders of reagent-grade WO_3_ (Merck KGaA, Darmstadt, Germany), H_3_BO_3_ (SIGMA-ALDRICH, St. Louis, MO, USA), La_2_O_3_ (SIGMA-ALDRICH, St. Louis, MO, USA), and Eu_2_O_3_ (SIGMA-ALDRICH, St. Louis, MO, USA) as starting materials. The homogeneous batch (10 g) has been melted for 20 min in air in a platinum crucible at 1240 °C. The glass was obtained by press quenching between two copper plates (cooling rate 10^2^ K/s). To prepare the glass-ceramics (GCs), the precursor glass 52WO_3_:22B_2_O_3_:26La_2_O_3_:0.5Eu_2_O_3_ was subjected to heat treatment at 680 °C for 3 h, 6 h, 9 h, 15 h, 18 h, and 21 h.

### 3.2. Samples Characterization

The glass transition (T_g_) and crystallization (T_c_) temperatures of the glass were determined using differential thermal analysis (Setaram, Labsys Evo 1600, Caluire-et-Cuire, France). The heating rate was 10 K/min in air atmosphere under an air flow of 20 mL/min. X-ray powder patterns of crystallized La_2_W_2_O_9_ glass ceramics were collected on a Bruker D8 Advance diffractometer, Karlsruhe, Germany equipped with a CuKa X-ray source. Monochromatization of the diffracted beam was achieved using a nickel filter in front of a Bruker LynxEye silicon-strip detector. During data collection, a spinner with a rotation speed of 15 rpm was used to average the measurement over the entire volume of the sample. The powder patterns were measured in the angular range of 5.5–80 degrees 2theta with a step of 0.04 degrees 2theta and an acquisition time of 0.4 sec/step per one detector channel. The phase composition of the samples was determined using the Match! v.3.16 program [41] and the ICDD PDF-2 database (2021) [42] as referent files. The program Topas v.4.2 [43] and Pawley fit procedure were used to determine the crystallite size and unit cell parameters of the collected patterns. The TEM observations were carried out using a transmission electron microscope JEM 2100 (JEOL, Tokyo, Japan) with a GATAN Orius 832 SC1000 CCD camera (AMETEK, Berwin, PA, USA) at an accelerating voltage of 200 kV. The specimen for TEM investigation was prepared by grinding the sample in an agate mortar and then disintegrating it in the form of an ethanol suspension by ultrasonic treatment for 6 min. A droplet of the suspension was coated on a standard carbon film on a Cu grid. The size distribution of the particles was performed with the image-processing program ImageJ, and the measurements of the interplanar distances were performed with the specialized software Digital Micrograph (Version 2.31.734). Photoluminescence (PL) excitation and emission spectra at room temperature for all studied samples were measured with a FluoroLog3-22 spectrofluorometer, 2014 (Horiba Jobin-Yvon, Longjumeau, France).

## 4. Conclusions

Glass-ceramic materials with enhanced photoluminescence emissions were obtained by controlled crystallization of the 52WO_3_:22B_2_O_3_:26La_2_O_3_:0.5Eu_2_O_3_ glass sample. Structural characterizations, i.e., TEM and XRD, verify the crystalline nature of the resulting glass-ceramics. X-ray studies show that during the heat treatment of the glass, the main crystalline phases are the La_2_W_2_O_9_ cubic phase and orthorhombic La_2_WO_6_. Morphological studies revealed that the sample contains spherical and rectangular nanosized particles. The majority of the particles are in the range of 20–40 nm. Photoluminescence spectra revealed that the obtained glass-ceramics can emit red light under excitation at 392 nm originating from the dominant dipole transition ^5^D_0_ → ^7^F_2_ of Eu^3+^ ions. Based on the results from the emission spectra, it was established that the formation of La_2_W_2_O_9_:Eu^3+^ nanocrystals and the appropriate degree of crystallinity are decisive factors for improving the luminescence properties of the samples. Formation of a second crystalline phase, La_2_WO_6_, leads to a drop in the emission intensity, because of the redistribution of Eu^3+^ ions in different crystalline phases. The optical properties confirm that the obtained glass-ceramics are suitable hosts for the incorporation of Eu^3+^ ions and have potential applications in the field of red light-emitting diodes.

## Figures and Tables

**Table 1:** Unit cell parameters and crystallite size of La_2_W_2_O_9_ cubic phase (SG. P2_1_3) after thermal treatment at 680 °C.

Crystallized at 680 °C	Unit Cell Parameter [Å]	Crystallite Size [nm]
3 h	7.134(1)	27.8(6)
6 h	7.118(2)	17.9(5)
9 h	7.132(1)	26.6(4)
12 h	7.131(1)	23.9(3)
15 h	7.1187(2)	22.1(3)
18 h	7.117(1)	22.1(4)
21 h	7.119(1)	23.3(4)

**Table 2:** Relative luminescent intensity ratio (R) of the two transitions (^5^D_0_ → ^7^F_2_)/(^5^D_0_ → ^7^F_1_) for 52WO_3_:22B_2_O_3_:26La_2_O_3_:0.5Eu_2_O_3_ glass and glass-ceramics heat treated at different time durations and of other reported Eu^3+^-doped oxide glasses and glass-ceramics.

Glass Composition	Relative Luminescent Intensity Ratio, R	Reference
Glass 52WO_3_:22B_2_O_3_:26La_2_O_3_:0.5Eu_2_O_3_	4.88	Current work
GC-3 h	4.93	Current work
GC-6 h	5.58	Current work
GC-9 h	5.83	Current work
GC-12 h	5.88	Current work
GC-15 h	5.95	Current work
GC-18 h	6.12	Current work
GC-21 h	5.92	Current work
Glass 50ZnO:(50-x)B_2_O_3_: xNb_2_O_5_:0.5Eu_2_O_3_:, x = 0, 1, 3 and 5 mol%	4.31–5.16	[29]
Glass 50ZnO:40B_2_O_3_:10WO_3_:xEu_2_O_3_ (0 ≤ x ≤ 10)	4.54 ÷ 5.77	[30]
Glass 50ZnO:40B_2_O_3_:5WO_3_:5Nb_2_O_5_:xEu_2_O_3_ (0 ≤ x ≤ 10)	5.09 ÷ 5.76	[31]
Glass 66P_2_O_5_–10.5Al_2_O_3_–3.05BaO–16.5K_2_CO_3_–0.7NaF–xEu_2_O_3_–0.5Nd_2_O_3_–(2.75-x) La_2_O_3_ (mol.%), where x = 0, 0.25, 0.5, 0.75, 1.5 and 2	3.72	[32]
Glass-ceramic 66P_2_O_5_–10.5Al_2_O_3_–3.05BaO–16.5K_2_CO_3_–0.7NaF–xEu_2_O_3_–0.5Nd_2_O_3_–(2.75-x) La_2_O_3_ (mol.%), where x = 0, 0.25, 0.5, 0.75, 1.5 and 2	4.72	
Glass 50ZnO:47B_2_O_3_:3Nb_2_O_5_:0.5Eu_2_O_3_	5.16	[33]
Glass-ceramic 50ZnO:47B_2_O_3_:3Nb_2_O_5_:0.5Eu_2_O_3_	5.21–5.49	
Glass 74.0 TeO_2_+25.0 Li_2_CO_3_+1.0 Eu_2_O_3_	3.70	[34]
Glass-ceramic 74.0 TeO_2_+25.0 Li_2_CO_3_+1.0 Eu_2_O_3_	3.65	
Glass 50Li_2_O·45B_2_O_3_·5Al_2_O_3:_ 2Eu_2_O_3_	3.91	[35]
Glass ceramic 50Li_2_O·45B_2_O_3_·5Al_2_O_3:_ 2Eu_2_O_3_	4.047	
Glass 7SiO_2_-47.4CaO-40.5Al_2_O_3_-4.1MgO-1Eu2O_3_	4.58	[36]
Glass-ceramic 7SiO_2_-47.4CaO-40.5Al_2_O_3_-4.1MgO-1Eu_2_O_3_	1.97	

**Table 3:** CIE chromaticity coordinates of 52WO_3_:22B_2_O_3_:26La_2_O_3_:0.5Eu_2_O_3_ glass and glass-ceramics.

Glass Composition	Chromaticity Coordinates (x, y)
Glass 52WO_3_:22B_2_O_3_:26La_2_O_3_:0.5Eu_2_O_3_	0.629, 0.328
GC-3 h	0.650, 0.343
GC-6 h	0.648, 0.345
GC-9 h	0.651, 0.345
GC-12 h	0.651, 0.345
GC-15 h	0.650, 0.346
GC-18 h	0.650, 0.346
GC-21 h	0.651, 0,346
NTSC standard for red light	0.670, 0.330
Y_2_O_2_S:Eu^3+^	0.658, 0.340

## Data Availability

Data are contained within this article.
